# Benchmarking the Covid-19 pandemic across countries and states in the USA under heterogeneous testing

**DOI:** 10.1038/s41598-021-94663-x

**Published:** 2021-07-26

**Authors:** Kenzo Asahi, Eduardo A. Undurraga, Rodrigo Wagner

**Affiliations:** 1 Escuela de Gobierno, Pontificia Universidad Católica de Chile, Av. Vicuña Mackenna 4860, Macul CP, 7820436 Santiago, Región Metropolitana Chile; 2 Centre for Sustainable Urban Development (CEDEUS), Santiago, Chile; 3 Millennium Initiative for Collaborative Research in Bacterial Resistance (MICROB-R), Santiago, Chile; 4 Research Center for Integrated Disaster Risk Management (CIGIDEN), Santiago, Chile; 5 CIFAR Azrieli Global Scholars Program, CIFAR, Toronto, Canada; 6 Business School, Universidad Adolfo Ibáñez, Santiago, Chile; 7 Center for International Development, Harvard, Cambridge, USA

**Keywords:** Diseases, Infectious diseases, Viral infection, Diagnosis, Health policy, Public health, Preventive medicine

## Abstract

Scientists and policymakers need to compare the incidence of Covid-19 across territories or periods with various levels of testing. Benchmarking based on the increase in total cases or case fatality rates is one way of comparing the evolution of the pandemic across countries or territories and could inform policy decisions about strategies to control coronavirus transmission. However, comparing cases and fatality rates across regions is challenging due to heterogeneity in testing and health systems. We show two complementary ways of benchmarking across territories and in time. First, we used multivariate regressions to estimate the test-elasticity of Covid-19 case incidence. Cases grow less than proportionally with testing when assessing weekly changes or looking across states in the USA. They tend to be proportional or even more than proportional when comparing the month-to-month evolution of an average country in the pandemic. Our results were robust to various model specifications. Second, we decomposed the growth in cases into test growth and positive test ratio growth to intuitively visualize the components of case growth. We hope these results can help support evidence-based decisions by public officials and help the public discussion when comparing across territories and in time.

## Introduction

The SARS-CoV-2 pandemic has imposed an enormous global burden, with about 140 million reported cases and 3.0 million deaths in 192 countries and territories, as of April 15, 2021^1^. Substantial evidence shows the disease burden of Covid-19 (illness caused by SARS-CoV-2) is higher than ascertained^2,3^. Public health officials have had to make urgent decisions about public health and social interventions to reduce the potential impact of Covid-19 with limited available evidence^4–8^. They have to continuously assess and adapt their decisions based on available resources, disease surveillance, and emerging scientific evidence^9–12^. The World Health Organization (WHO)^13^, governments^14–16^, and academic institutions^17–19^, have developed guidelines or suggested specific epidemiological and public health indicators (e.g., infection rates, case incidence, testing, health system capacity). These indicators are meant to support public health officials make decisions about the timing and intensity of non-pharmaceutical strategies to contain or mitigate the spread of the virus.

Benchmarking based on the increase in total cases or crude case fatality rates, the ratio between cumulative Covid-19 deaths and cases in the same period is one way to compare performance across states, regions, or countries. Comparing case fatality rates is challenging. They are typically affected by right censoring (time delay between the onset of symptoms and death) and under-ascertainment (mild Covid-19 cases or asymptomatic SARS-CoV-2 infections often go undetected)^2,20,21^. On the other hand, Covid-19 case counts depend on laboratory confirmation, so the number of reported cases is a function of testing. As epidemiologists have noted^22–24^, epidemic curves of reported cases are influenced by diagnostic testing capacity. Therefore, benchmarking may be of limited use because countries or even subnational administrative entities, such as states within the USA, do not have the same testing policies or test availability for SARS-CoV-2^6^. This relation between testing capacity and Covid-19 cases has generated confusion and sparked controversies between experts and public officials in many countries^25–29^.

Ideally, public health officials would carry out testing based on representative random samples of the population (e.g., national, state) to estimate infection rates and case-fatality rates. Most countries and states do not offer unlimited testing for Covid-19, despite enormous increases in global testing capabilities^30^. However, testing is far from random. At the early stages of an epidemic caused by a novel emerging infectious disease, such as SARS-CoV-2, surveillance is typically biased towards the more severe cases because of limited diagnostic capabilities^3^. Limited resources usually mean more stringent inclusion criteria for testing, including, for example, a combination of clinical symptoms and travel history. These restrictive criteria would often result in a higher proportion of positive tests and miss SARS-CoV-2 infections that do not comply with testing requirements. Another scenario of limited testing capabilities may be a surge in Covid-19 cases that rapidly increases testing demand, typically increasing test positivity. The WHO recommends a proportion of positive Covid-19 tests below 5% as one indicator of epidemic control^13^. Differences in testing need to be taken into account when benchmarking and communicating the evolution of the pandemic. A purist would argue that one cannot compare countries or territories with different levels of testing without random sampling.

Here we take a pragmatic approach to suggest two relatively simple ways of comparing total Covid-19 cases and case fatality rates across territories, such as countries or states in the USA, with varying degrees of Covid-19 testing. This approach is also helpful to benchmark changes over time in a given territory. The article has two primary analyses. First, we used multivariate regressions to estimate the test elasticity of Covid-19 incidence and case fatality rate in the early stages and during the first year of the pandemic. The test elasticity shows the relationship between testing of Covid-19 and reported cases or case fatality rate (in logarithms) in a context with limited testing. In the short-term (weekly changes), increases in test supply imply testing healthier people (i.e., an elasticity smaller than one and greater than zero). In the long term, testing supply should adjust to increases in the demand for tests (i.e., case elasticity of tests around one). Second, we algebraically decomposed and visualized the growth in Covid-19 cases into growth in tests and the positive test ratio (PTR). This growth decomposition is a complementary way of benchmarking the evolution of cases that allows visualizing the source of change in reported Covid-19 cases. We hope that our results can help public health officials when they need to benchmark performance over time or vis-á-vis other territories.

## Materials and methods

### Test-elasticity

#### Early stages of the pandemic

We estimated the test-elasticity-of-Covid-19-incidence for countries globally (n = 42; limited to countries that report the number of lab tests)^31^ and US states (n = 51)^32^. We used a multivariate regression with the week-on-week change in the weekly lab-confirmed Covid-19 cases or case fatality rates as our dependent variable, using data from early April 2020. As independent variables, we used testing per capita, health expenditures, number of days since the 100th lab-confirmed Covid-19 case occurred until the last day of analysis, the share of the population ≥ 70 years of age with chronic respiratory disease. We used a limited number of covariates in our analysis because we had a few observations. Health expenditures per capita are an essential characteristic of health systems as they reflect, at the minimum, the purchase of medicines and supplies, building and maintenance of health infrastructure (e.g., facilities, laboratories), and costs related to health workers^33^. We used the number of days since the 100th lab-confirmed case occurred to adjust for the relative time of the epidemic in each location (US state or country). Last, we included the share of the population older than 70 years of age with chronic respiratory disease, including chronic obstructive pulmonary disease and asthma. The population structure varies between locations, and individuals older than 70 years of age with underlying conditions are at a higher risk of death than the general population^3,34,35^. Variable definitions are shown in the Supplementary Material, Table S1.

We used multivariate regression to estimate the test elasticity of case incidence. The regression specification is:

1 $${\Delta ln \, (Y}_{i})= \alpha + \beta \cdot \Delta ln \,({Test}_{i})+ {\sum \delta }_{k}{\cdot X}_{k,i}+ {\varepsilon }_{i}$$

where the subscript (*i*) stands for country or state, and (*k*) for covariates included. *Y* is the dependent variable (Covid-19 cases or case fatality rate) and *Test* is the independent variable of interest, the amount of negative and positive tests informed in the country or state *i*. Because we used logarithms for *Y*_*i*_ and *Test*_*i*_, *β* captures the test elasticity of Covid-19 case incidence, controlling for a vector of covariates *X*_*k*_ (*k* stands for days since 100 cases, health expenditures per capita, and proportion of the population older than 70 years of age with chronic respiratory disease, including chronic obstructive pulmonary disease and asthma; Table S1). ε is an error term. In other words, *β* allows us to compare the evolution of cases for two countries with different levels of testing. Note that by first differencing both the dependent and the key variable, *β* accounts for any unobserved and time-invariant effect (e.g., income, quality of healthcare, age distribution) in a territory that may impact the level of testing and the level of the disease. Still, we kept covariates as controls to test their impact on *β*.

#### Medium to long-term testing

To test whether the elasticity has changed during the pandemic, we use data from April to December 2020 and compute panel regressions with fixed effects by geography (countries or US states) ${\mu }_{i}$ and period ${\lambda }_{t}$, to estimate the elasticity $\beta$ using the regression model:

2 $$\Delta \mathrm{ln} \, {y}_{i,t}= \beta \mathrm{\Delta ln} \, {Tests }_{i,t}+{\mu }_{i} +{\lambda }_{t} +{\epsilon }_{i,t}$$

where $\mathrm{\Delta ln} \, {y}_{i,t}$ is the log change in outcomes like cases and deaths; while $\mathrm{\Delta ln} \, Tests$ is the change in tests. We conducted this analysis using weekly and monthly data.

### Case growth decomposition

A complementary way of benchmarking the evolution of cases vis-à-vis other periods or territories with heterogeneous testing strategies is to use a simple algebraic decomposition. Because weekly cases can be decomposed as the multiplication of total tests ${T}_{week}$ and PTR (${Cases}_{week}/Test{s}_{week}$), transforming to logarithms and taking the difference over weeks yields that the growth of cases can be decomposed as

3 $$\Delta \mathrm{ln} \, Cases=\Delta \mathrm{ln} \, Test+\Delta \mathrm{ln} \, PTR$$

This growth decomposition is helpful to visualize the source of change in reported cases for comparison across countries or states in the USA.

## Results

### Test-elasticity

#### Early stages of the pandemic

**Figure 1:**
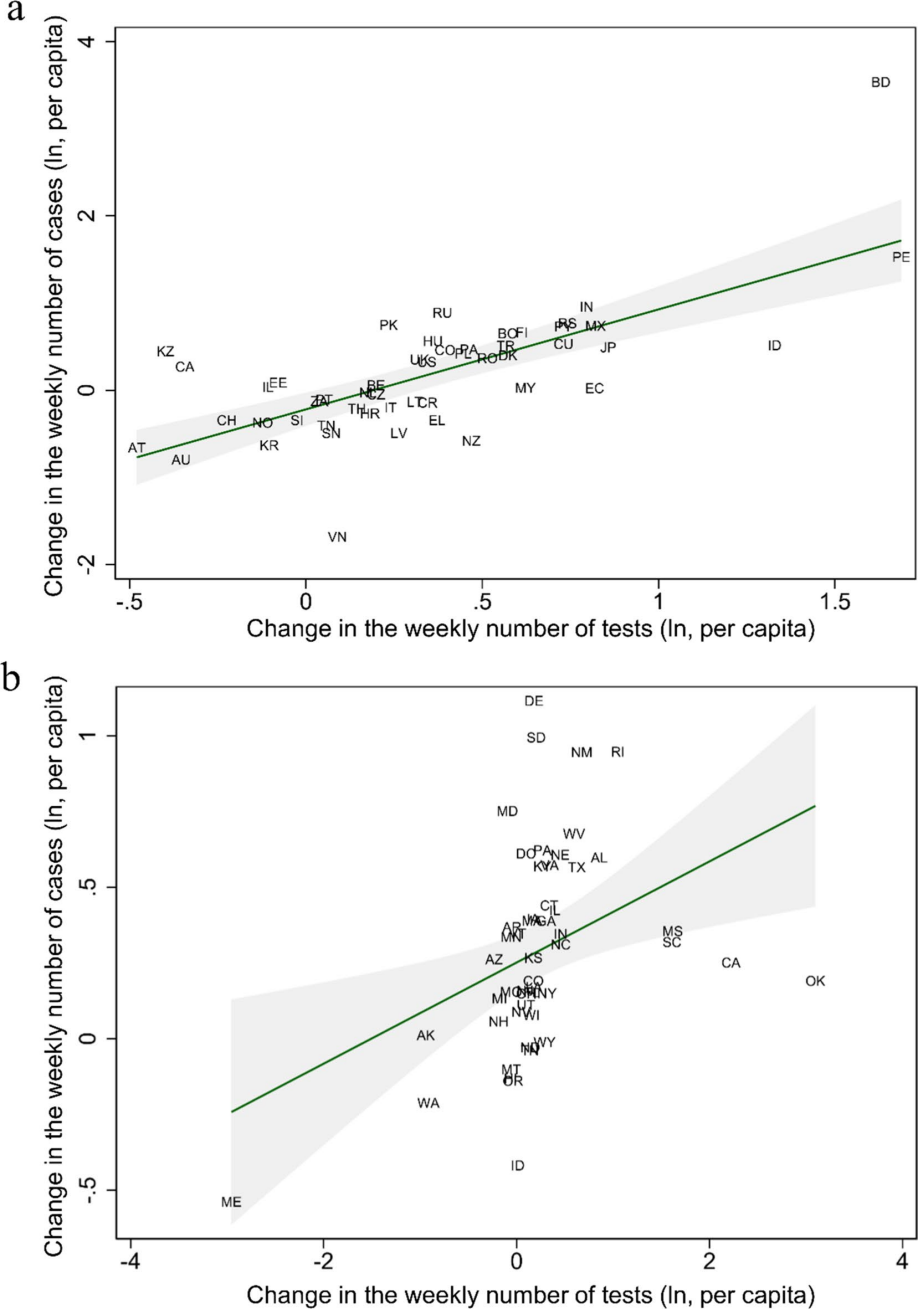
Changes in Covid-19 cases per capita relative to changes in the number of tests for countries and states in the USA. Panels show the growth in weekly cases and tests (in logarithms of the per capita rate) for (**A**) countries and (**B**) States in the USA that have reported Covid-19 cases. Data compares the week ending April 10th, 2020, with the previous week. (**A**) β = 0.86; 95% CI 0.57–1.15; p < 0.001; (**B**) β = 0.17; 95% CI 0.03–0.31; p = 0.02. The Supplementary material shows a higher elasticity for (**B**) if we restrict logarithmic week-on-week changes of testing rates between − 1 and + 1.5, the range observed in the global sample. In Supplementary Material, Figure S1: β = 0.54, p < 0.0001, 95%CI: 0.36–0.71. Names and abbreviations for countries and US States are included in the Supplementary Material’s Table S5.

Figure 1 shows an unadjusted estimate of the test-elasticity of cases; the association between logarithmic changes in testing per capita and reported cases. Figure 1A shows a consistent association between the increase in testing per capita and reported Covid-19 cases by country (β = 0.86; 95% CI 0.57–1.15; p < 0.001). In other words, a 10% increase in testing was associated with a ~ 9% increase in reported cases. Considering that a test elasticity = 1 was included in the 95% confidence interval, we cannot reject that growth in weekly cases was fully proportional to the increase in testing. By contrast, Fig. 1B shows that the test-elasticity of cases was substantially lower (β = 0.17; 95% CI 0.03–0.31; p = 0.02) for states in the USA. This result suggests that the evolution of Covid-19-case-growth was primarily driven by changes in PTR rather than testing. We get larger elasticities, more comparable to the global sample, when excluding outlier states in the USA and focusing on the same range of testing growth (β = 0.54, p < 0.0001, 95% CI 0.36–0.71; Supplementary Figure S1). The difference in the estimated elasticity of the cross-country sample (Fig. 1A) relative to US states (Fig. 1B) stems from the fact that the US had a much broader variation in terms of test growth during the period evaluated. For instance, California and Oklahoma displayed substantial increases beyond two log points in testing. In contrast, states like Alaska and Maine experienced substantial decreases in testing during the week plotted.

Results in Table 1 are consistent with the findings in Fig. 1A for the cross-country analysis. With and without regression controls, we get point estimates for the elasticity around 0.86–0.88 (p-value < 0.001; columns 1 through 3). A 10% increase in testing was associated with about a 9% (95% CI 4.2–13.4%; p < 0.001) increase in reported cases. Moreover, changes in testing explained about 40% of the variance in cases for our regressions (r^2^ = 0.41), and adding health expenditures, population, days since the 100^th^ Covid-19 case, and share of the population ≥ 70 years of age with chronic respiratory disease added little explanatory strength to the model (r^2^ = 0.46).

**Table 1:** Global regression estimates for the change in Covid-19 cases and case fatality rate reported on the change in tests conducted by country in a week.

	(1)	(2)	(3)	(4)	(5)	(6)
	Cases^a^ (ln)	Cases^a^ (ln)	Cases^a^ (ln)	Fatality^b^ (ln)	Fatality^b^ (ln)	Fatality^b^ (ln)
Testing ^c^ (ln)	0.864***	0.869***	0.881***	− 0.869***	− 0.884***	− 0.893***
	(0.145)	(0.156)	(0.225)	(0.124)	(0.125)	(0.166)
Days since 100 cases ^d^		0.00115	− 0.0157		− 0.00419	− 0.0198
		(0.00719)	(0.0119)		(0.00716)	(0.0123)
Health expenditure per capita (USD, ln)			0.148			0.150
			(0.171)			(0.132)
Aged 70 + with a chronic respiratory disease (ln) ^e^			0.0796			0.0202
			(0.134)			(0.195)
Population (ln)			0.00573			0.0696
			(0.120)			(0.194)
Constant	− 0.193*	− 0.225	− 2.023	0.607***	0.726**	− 1.415
	(0.0912)	(0.258)	(1.526)	(0.0762)	(0.243)	(1.852)
Observations	42	42	42	38	38	38
R-squared	0.408	0.408	0.456	0.452	0.456	0.498

*p < 0.05, **p < 0.01, ***p < 0.001. Robust standard errors in parentheses. ln stands for natural logarithm.

^a^Confirmed cases of Covid-19″ during the week ending April 10th, 2020.

^b^Case fatality rate is the ratio between deaths due to Covid-19 and cases in the same period.

^c^Testing is the amount of negative and positive tests informed to each country’s health authority in the same period of cases^30^.

^d^The number of days since the cumulated number of cases was equal to or greater than 100^1^.

^e^Share of the population aged 70 or older with a chronic respiratory disease, including chronic obstructive pulmonary disease and asthma.

We obtained similar results for the case fatality rate but in the opposite direction (Table 1, columns 4 through 6). We found a test-elasticity-of-case-fatality slightly above minus one (β = -0.89; column 6). A 10% increase in testing yielded about 9% (95% CI − 12.2 to − 5.6%; p < 0.001) decrease in case fatality rates, with covariates adding limited explanatory strength (r^2^ changes from 0.45 to 0.50). We reran the regression with the following week (April 11th through April 17th, 2020) to test robustness. The results were largely consistent. We found an elasticity for reported cases of β = 0.77 (95% CI 0.23–1.30; p = 0.01; r^2^ = 0.43, column 3), and β = − 0.77 (95% CI − 1.40 to − 0.14; p = 0.02, r^2^ = 0.18, column 6) for case fatality rates (Supplementary Table S2).

Table 2 shows results across states in the USA. As in Fig. 1B, the magnitude of the elasticity was smaller for reported cases (β = 0.18) and case fatality rate (β = − 0.16). A 10% increase in testing yielded about 2% (95% CI 0.1–3.4%; p = 0.03, r^2^ = 0.25, column 3) increase in reported cases, and ~ 2% (95% CI − 3.1 to − 0.2; p = 0.03, r^2^ = 0.14, column 6) decrease in case fatality rates (only significant with controls). R^2^ was smaller for regressions comparing US states than countries globally. Elasticities for US states using the data from April 11th through April 17th, 2020, showed qualitatively similar results. We found a test elasticity of β = 0.17 (95% CI 0.08–0.27; p < 0.001, r^2^ = 0.29, column 3) for reported cases, and β = − 0.15 (95% CI − 0.30–0.01; p = 0.06, r^2^ = 0.12, column 6) for case fatality rates, though the latter was not significant at α = 0.05 (Supplementary Table S3). As an additional robustness analysis, we reran the regressions using the share of the population above 70 years of age as a covariate (Supplementary Table S4); our results held.

**Table 2:** USA regression estimates for the change in Covid-19 cases and case fatality rates reported by tests conducted by state in a week.

	(1)	(2)	(3)	(4)	(5)	(6)
	Cases^a^ (ln)	Cases^a^ (ln)	Cases^a^ (ln)	Fatality^b^ (ln)	Fatality^b^ (ln)	Fatality^b^ (ln)
Testing ^c^ (ln)	0.167*	0.169*	0.177*	− 0.0805	− 0.0832	− 0.164*
	(0.0700)	(0.0715)	(0.0810)	(0.0413)	(0.0467)	(0.0722)
Days since 100 cases ^d^		− 0.00897	− 0.0147		0.0147	− 0.0210
		(0.00750)	(0.00913)		(0.0120)	(0.0215)
Health expenditure per capita (USD, ln)			0.643*			0.00631
			(0.274)			(0.403)
Aged 70 + with a chronic respiratory disease (ln) ^e^			− 0.0131			− 0.298
			(0.182)			(0.300)
Population (ln)			0.0650			0.270
			(0.0686)			(0.166)
Constant	0.252***	0.433*	− 6.268	0.434***	0.129	− 4.311
	(0.0445)	(0.180)	(3.177)	(0.0782)	(0.296)	(4.849)
Observations	51	51	51	47	47	47
R-squared	0.154	0.177	0.250	0.0167	0.0430	0.140

*p < 0.05, **p < 0.01, ***p < 0.001. Robust standard errors in parentheses. ln stands for natural logarithm.

^a^Confirmed cases of Covid-19″ during the week ending April 10th, 2020.

^b^Case fatality rate is the ratio between deaths due to Covid-19 and cases in the same period.

^c^Testing is the amount of negative and positive tests informed to each country’s health authority in the same period of cases^30^.

^d^The number of days since the cumulated number of cases was equal to or greater than 100^1^.

^e^Share of the population aged 70 or older with a chronic respiratory disease, including chronic obstructive pulmonary disease and asthma.

#### Medium to long-term testing

Using weekly changes (Table 3, columns 3 to 4), the estimated elasticities in the global regressions (Panel A) are between 0.53 and 0.62. A 10% increase in testing is associated with an expected increase in 5–6% cases. In other words, you would expect around half of the extra cases if the relation were fully proportional. Comparing the number of cases across different countries or periods (e.g., against last week) on proportional terms (i.e., β = 1) does not reflect the average behavior observed in our data. Table 3 shows two additional findings. First, over time, the weekly elasticities are qualitatively similar in being less than proportional. For instance, until July 2020, the fixed-effects test elasticity of cases at the country level is 0.62 (Table 3, Panel A, Column 3). In the weeks between August and December 2020, performing the same calculation shows a slightly lower point estimate of 0.53 (Panel A, column 4). While statistically different at normal levels vis-à-vis the first months of the pandemic, it is still qualitatively similar to our story. Overall, we get a fixed-effects test elasticity of cases between 0.53 and 0.62 on weekly changes.

**Table 3:** Panel regressions with fixed effects by geography and period in 2020.

Dependent variable: log changes in cases	Monthly changes		Weekly changes	
	Until July	Aug-Dec	Until July	Aug-Dec
	(1)	(2)	(3)	(4)
**Panel A: global regressions**				
Testing (ln)	1.28***	1.38***	0.62***	0.53***
	(0.17)	(0.09)	(0.04)	(0.04)
Observations	250	430	1549	1809
R-squared	0.63	0.58	0.35	0.21
**Panel B: US states regressions**				
Testing (ln)	0.80***	0.62***	0.11***	0.04**
	(0.17)	(0.11)	(0.02)	(0.01)
Observations	168	275	1044	1145
R-squared	0.53	0.46	0.61	0.24

* *p* < 0.05, **p < 0.01, ***p < 0.001. Robust standard errors in parentheses. ln stands for natural logarithm. Because we control for fixed-effects in all regressions, all observed and unobserved invariant countries’ and US states’ characteristics are controlled for. Hence, we do not include covariates in these regressions.

Second, the effect is close to proportional or even more than proportional when analyzing monthly changes. The fixed-effects regressions for the first four months of the pandemic in Table 3, Column (1) show a higher than proportional effect, close to 1.3 (i.e., for every 10% increase in testing, we get close to 13% more cases). However, the point estimate for the elasticity in Column (2) is also around 1.3, but it has smaller standard errors than Column (1), being statistically larger than one. (See Supplementary Material for an extended discussion of elasticity in different time frames).

### Case growth decomposition

#### Early stages of the pandemic

**Figure 2:**
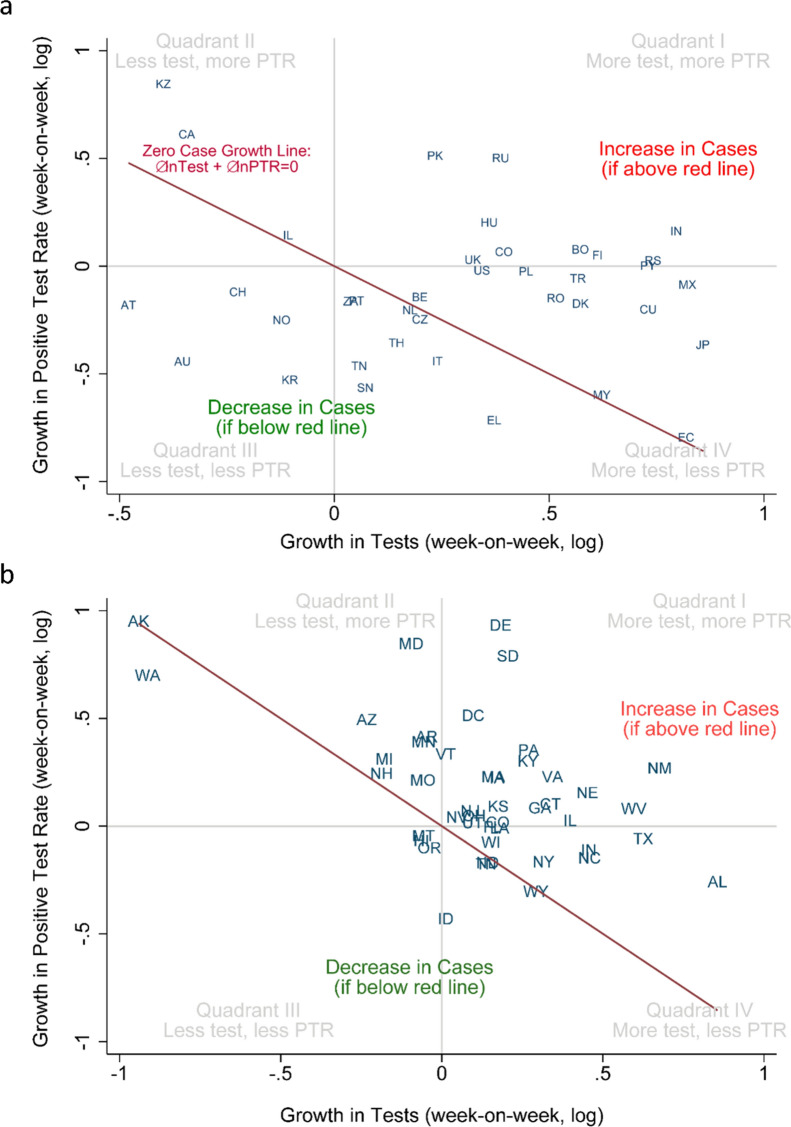
Growth Decomposition of Cases: Testing Growth vs. PTR Growth. Week-on-week growth of Testing and PTR across countries and US states. Points are countries (**A**) or US states (**B**). In both panels, we plot the growth of tests and PTR between the week ending April 10th and the previous week. The functional form of the previously mentioned growth is the change in the natural logarithm of per capita rates per week. The growth decomposition of cases is the sum of the increase in testing (horizontal axis) and PTR growth (vertical axis). The line is not a regression but represents zero case growth as $\mathrm{\Delta ln} \, Cases=\mathrm{\Delta ln} \, Tests+\mathrm{\Delta ln} \, PTR=0$. While territories above the line have growing cases, territories below the line have decreasing cases. The four quadrants (I to IV) in gray show the various combinations of increasing or decreasing testing and PTR. Notably, quadrants (II) and (IV) include growing and declining cases, depending on the side of the zero-case growth line. For instance, in (**B**), KY appears with decreasing cases but is associated with fewer tests and more PTR. The opposite combination happened in (**A**) for Ecuador. For visual purposes, countries in A were restricted to a pop > 5 million people, and US states in (**B**) exclude WA. Countries are represented by ISO 3 codes and US states by two-digit codes. The supplementary material shows how the USA moved over several weeks in the cross-country plot. Names and abbreviations for countries and US States are included in the Supplementary Material.

Figure 2 shows a growth decomposition of Covid-19 cases for countries (A) and US states (B). The origin (0, 0) shows that both testing and PTR remained the same as in the previous period. Figure 2 shows four quadrants where countries would fall when they increased or decreased in either testing or PTR. If testing eligibility and the test remain unchanged, when both testing and PTR grow (quadrant I), the number of cases increases. Conversely, when testing and PTR decrease (quadrant III), the number of cases is declining. When countries or states move along the y-axis (change in PTR with a constant number of tests) or x-axis (change in tests with a constant PTR), a positive weekly change means more cases, and a negative weekly change means fewer cases. Nevertheless, for quadrants II and IV, the net effect in cases is not evident. To aid interpretation, we plotted a downward sloping line that represents the zero-case-growth (i.e., where $\mathrm{\Delta ln} \, {Test}_{week}+\mathrm{\Delta ln} \, {PTR}_{week}=0;$ hence $\mathrm{\Delta ln} \, {Test}_{week}=- \mathrm{\Delta ln} \, {PTR}_{week}$). Countries or states above the line increased case growth in the past week; those below the line have decreased case growth. Case growth in most countries moves in complex trajectories. The distance to the zero-case growth line is a visual clue for the overall increase in cases.

In Fig. 2A, we see that countries like Russia (RU) and Greece (EL) increased the number of tests performed between April 3rd and April 10th, 2020. However, because PTR increased in Russia and decreased in Greece, we can conclude, solely by looking at Fig. 2A, that Covid-19 cases increased in Russia and decreased in Greece between April 3rd and April 10th, 2020. On the other hand, countries like Ecuador (EC) and Israel (IL) appear on top of the line, meaning that growth in Covid-19 cases was approximately the same as in the previous week ($\mathrm{\Delta ln} \, {Cases}_{week}=0$). Notably, this null change in case growth is accounted for by different levels of testing. In the graph, Israel decreased reported testing but increased the positive rate in the same proportion. By contrast, Ecuador massively increased testing during that week with a declining PTR, leaving case growth unchanged. This decomposition helps track case growth visually across jurisdictions with heterogeneous testing.

#### Decomposing within-country changes in the long term

**Figure 3:**
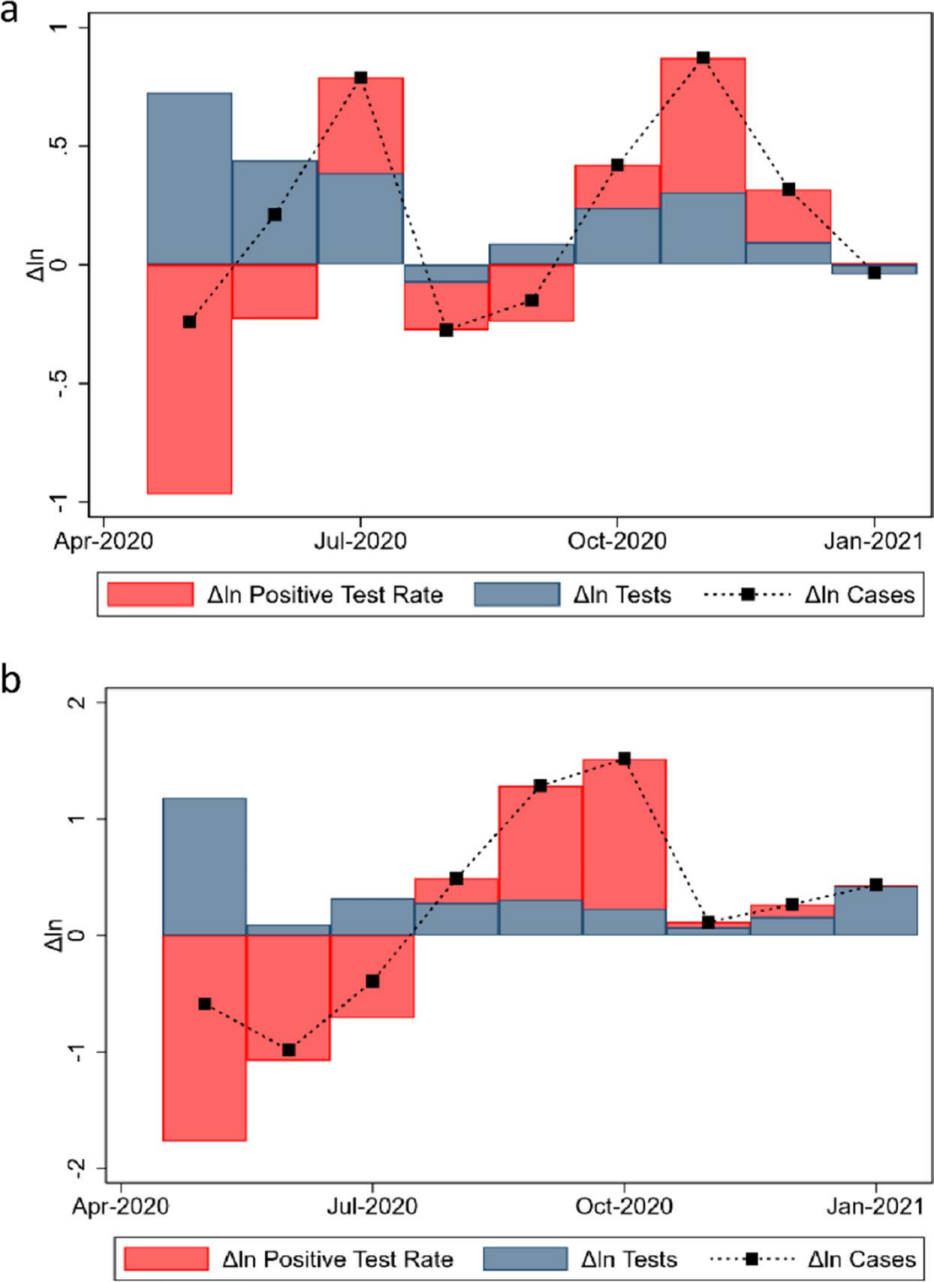
Exact decomposition of cases in the United States (**a**) and the United Kingdom (**b**).

Figure 3 shows the time series of the exact decomposition for the change in cases in (a) the USA and (b) the UK. Figure 3a displays the exact growth decomposition of Covid-19 cases in the USA using Eq. (3). When tests and PTR move with the same sign, the change in total cases, given by the small black squares, coincides with the stacked bars. When tests and PTR move in opposite directions, the difference in cases still corresponds to the sum of both effects, making the black squares appear inside the main bars. Two trends stand out. First, in around half of the months, PTR moved in the opposite direction of testing, coherent with a stronger relative ‘test supply’. Between October and December 2020, testing and PTR increased, as if the demand for tests were ahead of supply. The second point is that most of the month-to-month variations in cases in the graph are due to PTR changes rather than testing. Figure 3b displays the exact growth decomposition of Covid-19 cases in the UK (Eq. 3)$;$ a more explicit example where case growth can be accounted by PTR instead of testing.

**Figure 4:**
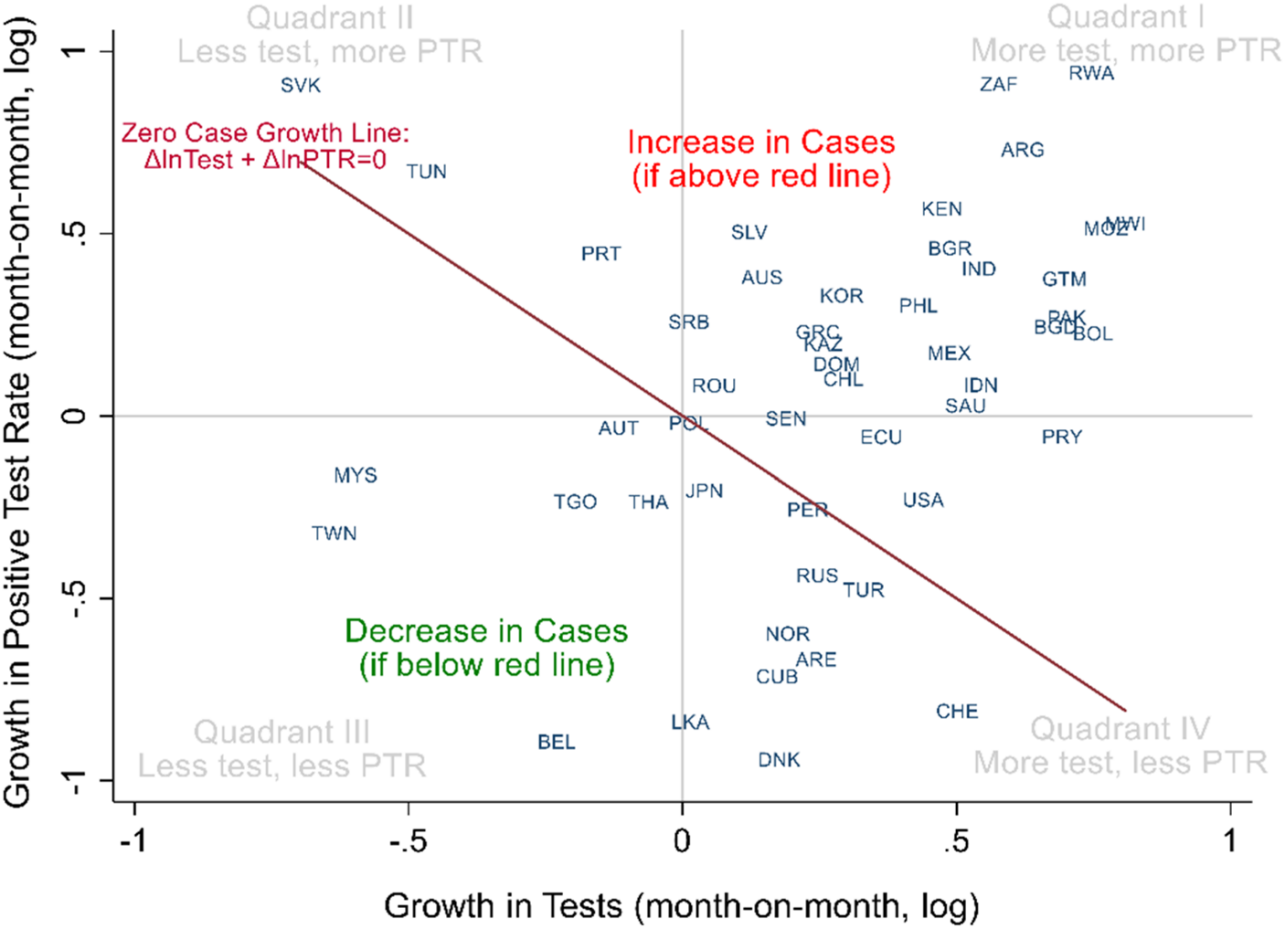
Exact decomposition of Covid-19 cases, month-on-month (growth in June 2020). The line is not a regression but represents the menu of possibilities of zero case growth. Names and abbreviations for countries and US States are included in the Supplementary Material.

Figure 4 displays the two components of the exact growth decomposition of Covid-19 cases (Eq. 3); month-on-month (growth in June 2020). The horizontal axis plots the growth of tests, and the vertical is the growth of PTR. The line is not a regression but represents a range of possible zero case growth. While territories above the downward sloping line have growing cases, territories below the line have decreasing cases. The four quadrants (I to IV) in gray show the various combinations of increasing or decreasing testing and PTR. Quadrants (II) and (IV) include growing and falling cases, depending on the side of the zero-case growth line.

To illustrate the interpretation of Fig. 4, we compare the USA and Portugal. Both countries displayed relatively similar and mild growth rates in cases during this month (June 2020). They are both close to and similarly above the red-line of zero-case growth. Their difference is the composition of that growth. While Portugal decreased testing and increased positivity (quadrant II), the plot shows the USA in precisely the opposite combination, of quadrant IV, with increased testing.

## Discussion

Public officials have often benchmarked Covid-19 cases under heterogeneous testing during the pandemic, comparing performance to other territories or vis-à-vis the sample territory in time (e.g., in a second or third epidemic wave). To illustrate, on June 23, 2020, former US President Donald Trump claimed that “cases are going up in the US because we are testing far more than any other country, and ever-expanding. With smaller testing, we would show fewer cases”^26^. One could use Fig. 3a to analyze this statement using a proportional logic. In June 2020, President Trump may have been at least partially correct. Had testing increased by zero in June, and had positivity been the same as plotted—something far from obvious– then cases would have had decreased instead of increased. In late July, Mr. Trump made a related statement, claiming that “cases are up because we have the best testing in the world and we have the most testing”^36^. Fig. 3a shows that this second statement was probably not accurate because the proportion of positive tests and the number of cases grew in the USA during July. Had the proportion of positive tests not changed with testing, we would have still observed a positive growth in overall Covid-19 cases, given by the height of the red bar in July. The months of October to December 2020 were qualitatively similar to July, with both testing and positivity growing in the USA.

Similar controversies about the relationship between testing and cases have occurred elsewhere and have been a source of misunderstandings during the pandemic^25–29^. The importance of examining cases and testing together is also shown by the existence of specific guidelines for health authorities to monitor test positivity rates based on specific thresholds^13,16,18,19,37^. As shown in our decomposition in Eq. (3), PTR and cases are not algebraically independent. In such context, our exact decomposition offers a parsimonious representation of this phenomenon. This decomposition could be used to gain additional insights to the relation of Covid-19 cases and testing, such as in Liang et al.^38^.

While the exact decomposition of Eq. (3) is always algebraically correct, thinking about the counterfactuals of how reported cases would have evolved  had testing been unchanged might require some additional assumptions. For example, it would require that the Tests and PTR, namely blue and red bars in Fig. 3, respectively, be orthogonal to each other. This is equivalent to saying that the regressions in Eq. (2) had a true elasticity $\beta =1$ (see Supplementary Appendix). Were $\beta$ smaller or larger than one means that tests and PTR growth have negative or positive covariances, respectively. $\beta =1$ means, for example, that a 10% increase in tests would be associated with a 10% increase in cases, as in the proportional case of Eq. (3). Nevertheless, our regression analysis suggests that this proportional thinking is not always an adequate comparison to what tends to happen on average. For instance, the weekly changes in an average country (Table 3) displayed elasticities of 0.5–0.6, meaning that a 10% test growth would be associated with a case growth of only 5–6% in an average country in an average week. Thus, a relevant remark is that analysts looking at weekly changes should be careful when extrapolating if cases grow less than proportional, despite more testing (i.e., that PTR decreases week-on-week). When benchmarking month-to-month changes (Table 3, columns 1 and 2), the average country moves proportional or even more robust than proportional in cases (i.e., $\beta >1$).

Our approach is a statistical way of benchmarking, reflecting what tends to happen on average between cases and testing. Our analysis does not constitute a causal story coming from exogenous variation in testing. Our methods could be readily generalizable to other territories or future epidemics. However, the estimated elasticities reflect a global average with substantial variations within countries. In particular, we show that the test-elasticity of Covid-19 case incidence is heterogeneous depending on the country’s level of human development (see the Supplementary Material’s p. 13).

## Conclusions

Public officials and analysts often compare the incidence of COVID-19 across territories or in time with heterogeneous testing. Our results show two relatively simple ways of benchmarking these quantities across regions or over time. We estimated test-elasticities of cases for different frequencies and geographies, showing that cases tend to grow less than proportional with testing when looking at weekly changes or looking across US states. Cases and testing tend to be proportional or even more than proportional when comparing the month-to-month evolution of an average country. We also offer an exact growth decomposition between test and PTR growth, mimicking our regressions when the effect is precisely proportional. Data required for our estimates are readily available to public health officials through Covid-19 data repositories; data and code for our analyses are also available. As the pandemic unfolds and more consistent data hopefully become available^39^, we hope these results can help support evidence-based decisions by public officials and help the public discussion when comparing across territories and in time.

## Supplementary Information

Supplementary Information.

## Data Availability

All data analyzed in this study are publicly available at the following sites: Centers for Medicare and Medicaid Services. Health expenditures by state of residence, 1991–2014. Centers for Medicare & Medicaid Services, Baltimore, MD. 2017. Available at: https://go.cms.gov/2KkMk0f. Accessed April 2021. European Centre for Disease Prevention and Control. Data on the geographic distribution of Covid-19 cases worldwide. eCDC, Solna, Sweden. 2020. Available at: https://bit.ly/2XWhCm2. Cross-country database of Covid-19 testing. Available at: https://github.com/owid/covid-19-data/tree/master/public/data/testing. Institute of Health Metrics and Evaluation. 2021. Global Health Data Exchange. IHME, University of Washington, Seattle, WA. Available at: http://ghdx.healthdata.org/. The Covid Tracking Project. State by state data and annotations. USA. 2021. Available at: https://covidtracking.com/data. Accessed April 2021. World Health Organization. Global Health Expenditure Database. WHO, Geneva. 2021. Available at: https://apps.who.int/nha/database. Accessed April 2021.
