# Supplementary material for: Benchmarking the Covid-19 pandemic across countries and states in the USA under heterogeneous testing
Source: Sci Rep. 2021 Jul 26;11:15199. doi: 10.1038/s41598-021-94663-x (PMC8313551; doi:10.1038/s41598-021-94663-x)
Supplement: Supplementary file 1 — Supplementary Information. [file 41598_2021_94663_MOESM1_ESM.pdf]

Scientific Reports

Electronic Supplementary Material

Benchmarking the COVID-19 pandemic across countries and the states in the U.S.A. under heterogeneous testing

Kenzo Asahi[1,2], Eduardo A. Undurraga[1,3,4,5*], Rodrigo Wagner[4,5]

[1] *Escuela de Gobierno, Pontificia Universidad Católica de Chile, Santiago, RM, Chile*

[2] *Centre for Sustainable Urban Development (CEDEUS), Chile*

[3] *Millennium Initiative for Collaborative Research in Bacterial Resistance (MICROB-R), Chile*

[4] *Research Center for Integrated Disaster Risk Management (CIGIDEN), Santiago, Chile*

[5] *CIFAR Azrieli Global Scholars program, CIFAR, Toronto, Canada*

[6] *Business School, Universidad Adolfo Ibáñez, Santiago, Chile*

[7] *Center for International Development, Harvard, Cambridge, USA*

Contents

A. Materials and methods 2

Clarifications about the interpretation of regression coefficients for elasticity 3

Estimated elasticity and frequency of changes 4

An alternative interpretation of β 6

Use of decomposition for the predictability of future cases 6

B. Additional results 7

Elasticity regression results 7

Case growth decomposition 11

Elasticities can vary by the country’s human development index 14

C. Names and abbreviations for countries and US States 15

References in the supplementary material 17

*Address for correspondence: Eduardo A. Undurraga; ORCID: 0000-0002-4425-1253. Av. Vicuña Mackenna 4860, Macul CP 7820436, Santiago, Región Metropolitana, Chile. Telephone: +56 (2) 23549536, email: eundurra@uc.cl

A. Materials and methods

Table S1. Definition of variables used in the regressions

**Variable name**	**Definition**	**Source**
COVID-19 cases	Lab-confirmed cases of COVID-19 reported to the surveillance system	1,2
Case fatality rate	The proportion of deaths from COVID-19 among all reported cases to the surveillance system	1,2
**Covariates**		
Testing	Cumulative number of RT-PCR negative and positive tests for SARS-CoV-2 informed to each country's health authority tests, in logarithms or change in logarithms	3
Days since 100 cases	Number of days since the cumulative number of lab-confirmed COVID-19 cases were ≥100	3
Health expenditures per capita	Health expenditures per capita (2019 US dollars)	4,5
Aged 70+ with a chronic respiratory disease	Share of the population older than 70 years of age with chronic respiratory disease, including chronic obstructive pulmonary disease and asthma, in logarithm	6,7
Population	Population size	7

Notes

Clarifications about the interpretation of regression coefficients for elasticity

Public Health officials worldwide are comparing various ratios and quantities that are not entirely independent of one another. Here, we briefly clarify the meaning of the coefficient we estimated for some of the policy-relevant amounts monitored.

If T_t and C_t are weekly tests and cases in week t, the positive test rate PTR is C_t / T_t.

The change in PTR can be decomposed as $\Delta\ ln\ [C_t/T_t] = \Delta\ ln\ C_t - \Delta\ ln\ T_t$; but the coefficient estimated in our paper says that $\Delta\ ln\ C_t = \beta\ \ \Delta\ ln\ T_t$; which replaced in the previous equation yields that $\Delta\ ln\ PTR\ = [\beta - 1]\ \ \Delta\ ln\ T_t$.

In general, looking at the structure of this latter formula, any $\beta < 1$ would imply a decrease in PTR when tests grow, while a $\beta > 1$ would mean a growing PTR. As an example, a $\beta \approx 0.2$ like the one we estimated in Figure 1b for US states would mean that a thought experiment of 10% change in tests ($\Delta\ ln\ T_t = 0.1$) would make $\Delta\ ln\ PTR = [0.2 - 1]\ \ [0.1] = -0.08$. So smaller by 8% of the original PTR. To get the new PTR, we have to multiply the initial ratio by one plus the above number. That is $[PTR]_{new} = [PTR]_{old}[1 + \Delta\ ln\ PTR]$= $[PTR]_{old}\ [\ 1 +\ \ [\beta - 1]\ \ \Delta\ ln\ T_t\ ]$. Following the previous numeric example and assuming the initial PTR was 15%, then the new PTR would be $0.15\ \times\ \ [1 + (-0.08)]\ = 0.138$ or 13.8%.

A final comment is that our estimate of β for the average US state or average country during the current period is not a constant written in stone, but it could undoubtedly evolve.

Summing up, the coefficient β estimated in the main section allows us to compare the evolution of cases for two countries with different levels of testing. Also, the estimated key coefficient helps to benchmark the change of the ratio of positive tests, which also depends on testing. This idea could complement the growth decomposition of cases, an analogous way to decompose case growth between testing intensity and PTR. The two methods are the same in the particular case when the test-elasticity is equal to one, and the R^2 of the regression is 100%.

Estimated elasticity and frequency of changes

To understand why a weekly elasticity might be smaller than the monthly elasticity, it is instructive to follow Hawawini (1983).[8] He deals with the change in the elasticity of two log-changes when periods change. Let's call $c_{t,T} \equiv \Delta lnCases_t$ and $x_{t,T} \equiv \Delta lnTests_t$.

The changes are made every T periods (i.e., T=7 is weekly, T=30 if monthly). In a contemporaneous regression, one estimates an elasticity β from

$$c = \beta x + \epsilon \,.$$

In Hawawini's (1983) spirit, one could assume that the actual process of generating the data on cases could be correlated with testing not only in the current period but also potentially with one lag and one lead.

To summarize how significant are these inter-temporal correlations in comparison to the contemporaneous correlation Hawawini defines a ratio

$$q_{c,x} \equiv \frac{\rho_{c,x(t-1)} + \rho_{c,x(t+1)}}{\rho_{c,x}}$$

For the auto-correlations of testing changes over time, one gets a similar

$$q_{x,x} \equiv \frac{\rho_{x,x(t-1)} + \rho_{x,x(t+1)}}{\rho_{x,x}} = 2\rho_{x,x(t-1)}$$

Then, Hawawini shows that the elasticity for a T period difference $\beta(T)$ relates to the elasticity using one-period differences, $\beta(1)$, through the following expression

$$\beta(T) = \beta(1)\ \frac{T + \ [T-1]\ q_{c,x}}{T + \ [T-1]\ q_{x,x}}$$

Taking derivatives of the difference with respect to the period T, one gets

$$\frac{\partial\beta(T)}{\partial T} = \beta(1)\ \frac{q_{c,x} - q_{x,x}}{\left[T + (T-1)q_{x,x}\right]^2}$$

This means that the elasticity $\beta(T)$ grows with the period of the difference considered, T, when the above derivative is positive. That is the case when $q_{c,x} > q_{x,x}$, making the numerator positive. In other words, the elasticity increases from weekly to monthly when cases c have relatively stronger lag-lead correlations with testing x, vis-à-vis the autocorrelation of testing.

$$\frac{\rho_{c,x(t-1)} + \rho_{c,x(t+1)}}{\rho_{c,x}} > 2\rho_{x,x(t-1)}$$

An alternative interpretation of β

A regression $\Delta \ln Cases = \beta \, \Delta \ln Tests + \epsilon$, to get the elasticity β, could be combined with our exact decomposition, which we replace on the left-hand side of the regression, leading to $\Delta \ln Tests + \Delta \ln PTR = \beta \, \Delta \ln Tests$. Collecting terms related to testing on the right-hand side yields an implicit regression of positivity on testing would be $\Delta \ln PTR = (\beta - 1) \, \Delta \ln Tests$. That is why in some regression Tables, we will display both the standard test to see whether the elasticity β is statistically different from zero, and also show a formal test of significance for $(\beta - 1)$. For example, if $(\beta - 1)$ were statistically zero, then it would be equivalent to claim that there is no significant correlation between changes in positivity and changes testing, validating the proportional approach in the exact decomposition used in Figure 3 and Figure 4, which is coherent to a $\beta = 1$.

Use of decomposition for the predictability of future cases

To predict future cases, we could run a dynamic regression of current case growth on the lagged PTR and testing growth. Specifically,

$$\Delta \ln cases_t = \alpha + \beta_{lagged\ test} \, \Delta \ln tests_{t-1} + \beta_{lagged\ PTR} \, \Delta \ln PTR_{t-1} \qquad \text{(R1.4)}$$

Given our exact decomposition, if β coefficients are the same, the previous equation would collapse to a standard first-order autocorrelation of case growth.

$$\Delta \ln cases_t = \alpha + \rho \Delta \ln cases_{t-1}$$

To illustrate the point, we can consider the case of India from April to Dec 2020. When predicting the growth in cases, the coefficient on the previous week's tests ($\Delta \ln tests_{t-1}$) is twice as large as the coefficient on lagged PTR (0.81 vs 0.42, p-value of difference = 0.05). Therefore, a 10% increase in testing would be associated with an 8% expected increase in cases next week. In contrast, a 10% increase in PTR would be associated with only a 4% expected increase in cases next week. Our exact decomposition therefore allows separating these two different predictions.

B. Additional results

Elasticity regression results

Figure S1 shows week change in cases and tests (in logarithms) US states, similar to Figure 1b in the article's main text, but keeping differences between -1 and + 1.5 log points.

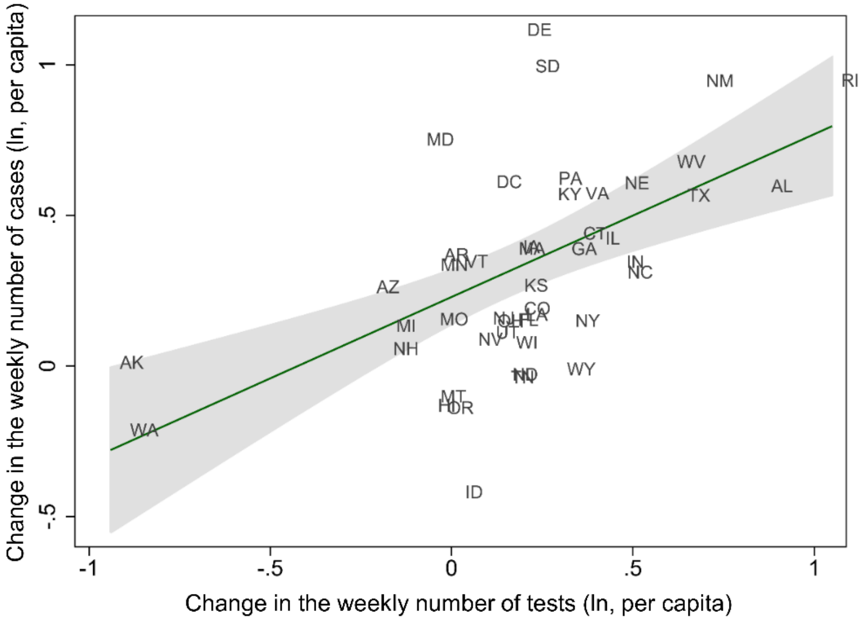

Figure S1. Change in cases per capita relative to change in the number of tests for states in the USA, excluding outliers and focusing on the same range of testing growth as the global cross country sample. Data shown are changes in weekly reported cases and testing between April 4 through April 10, 2020. Sample of US states restricted to logarithmic week-on-week changes of testing rates between -1 and +1.5, as observed in the global sample. In Figure S1: $\beta=0.54$, $p<0.0001$, 95%CI: 0.36–0.71.

Table S2. Global regression estimates for the change in COVID-19 cases and case fatality rates reported on tests conducted by each country in a week

	(1)	(2)	(3)	(4)	(5)	(6)
	Cases[a] (ln)	Cases[a] (ln)	Cases[a] (ln)	Fatality[b] (ln)	Fatality[b] (ln)	Fatality[b] (ln)
Testing [c] (ln)	0.982***	0.985***	0.766**	-0.688**	-0.792*	-0.769*
	(0.191)	(0.209)	(0.265)	(0.251)	(0.347)	(0.310)
Days since 100 cases [d]		0.000286	0.00243		-0.00891	-0.0214
		(0.00785)	(0.0131)		(0.0101)	(0.0193)
Health expenditure per capita (USD, ln)			-0.0166			0.124
			(0.113)			(0.184)
Aged 70+ with a chronic respiratory disease (ln) [e]			-0.142			0.0872
			(0.213)			(0.109)
Population (ln)			0.202			-0.0319
			(0.168)			(0.118)
Constant	-0.144*	-0.153	-1.617	0.374***	0.671	-0.451
	(0.0611)	(0.271)	(1.162)	(0.0962)	(0.415)	(1.633)
Observations	51	51	51	50	50	50
R-squared	0.347	0.347	0.428	0.131	0.149	0.183

Notes

* $p < 0.05$, ** $p < 0.01$, *** $p < 0.001$. Standard errors shown in parentheses. ln stands for natural logarithm.

[a] Confirmed cases of COVID-19 from March 22nd through April 4th, 2020 (14 days).

[b] Case fatality rate is the ratio between deaths due to COVID-19 and cases in the same period.

[c] Testing is the amount of negative and positive tests informed to each country's health authority in the same period of cases [3].

[d] Number of days since the cumulated number of cases was equal or greater than 100 [3].

[e] Share of the population aged 70 years or older with a chronic respiratory disease, including chronic obstructive pulmonary disease and asthma [6,7].

Table S3. USA regression estimates for the change in COVID-19 cases and case fatality rates reported on tests conducted by each state in a week

	(1)<br>Cases[a] (ln)	(2)<br>Cases[a] (ln)	(3)<br>Cases[a] (ln)	(4)<br>Fatality[b] (ln)	(5)<br>Fatality[b] (ln)	(6)<br>Fatality[b] (ln)
Testing [c] (ln)	0.140*<br>(0.0597)	0.147**<br>(0.0503)	0.174***<br>(0.0466)	-0.0819<br>(0.0667)	-0.0847<br>(0.0547)	-0.146<br>(0.0757)
Days since 100 cases [d]		-0.00883<br>(0.0134)	-0.0344<br>(0.0208)		0.00328<br>(0.0165)	0.0266<br>(0.0252)
Health expenditure per capita (USD, ln)			0.901**<br>(0.330)			-0.0849<br>(0.383)
Aged 70+ with a chronic respiratory disease (ln) [e]			-0.390<br>(0.230)			0.586<br>(0.372)
Population (ln)			0.186<br>(0.108)			-0.152<br>(0.151)
Constant	-0.0834<br>(0.0480)	0.167<br>(0.374)	-11.38**<br>(3.659)	0.295***<br>(0.0628)	0.202<br>(0.487)	4.638<br>(4.370)
Observations	44	44	44	44	44	44
R-squared	0.0917	0.112	0.290	0.0195	0.0213	0.116

Notes

* $p < 0.05$, ** $p < 0.01$, *** $p < 0.001$. Standard errors shown in parentheses. ln stands for natural logarithm.

[a] Confirmed cases of COVID-19" from March 22nd through April 4th, 2020 (14 days).

[b] Case fatality rate is the ratio between deaths due to COVID-19 and cases in the same period.

[c] Testing is the amount of negative and positive tests informed to each country's health authority in the same period of cases [3].

[d] Number of days since the cumulated number of cases was equal or greater than 100 [3].

[e] Share of the population aged 70 years or older with a chronic respiratory disease, including chronic obstructive pulmonary disease and asthma [6,7].

Table S4. Regression estimates for the change in Covid-19 cases and case fatality rate reported on the change in tests conducted by country in a week

	Global		USA	
	Cases[a] (ln)	Fatality[b] (ln)	Cases[a] (ln)	Fatality[b] (ln)
Testing [c] (ln)	0.941***	-0.921***	0.176*	-0.167*
	(0.256)	(0.179)	(0.0813)	(0.0728)
Days since 100 cases [d]	-0.0288*	-0.0191	-0.0148	-0.0195
	(0.0131)	(0.0105)	(0.0089)	(0.0210)
Health expenditure per capita (USD, ln)	0.0464	0.146	0.648*	0.0155
	(0.160)	(0.117)	(0.281)	(0.402)
Aged 70+ (ln) [e]	0.234	0.0672	-0.0482	-0.427
	(0.244)	(0.242)	(0.304)	(0.492)
Population (ln)	0.153*	0.102	0.0170	-0.171
	(0.0704)	(0.0712)	(0.279)	(0.393)
Constant	-1.763	-1.476	-6.381	-4.135
	(1.484)	(1.637)	(3.269)	(4.952)
Observations	50	46	51	47
R-squared	0.569	0.509	0.250	0.137

Notes. * $p < 0.05$, ** $p < 0.01$, *** $p < 0.001$. Robust standard errors are shown in parentheses. ln stands for natural logarithm.

[a] Confirmed cases of Covid-19" during the week ending April 10th, 2020.

[b] Case fatality rate is the ratio between deaths due to Covid-19 and cases in the same period.

[c] Testing is the amount of negative and positive tests informed to each country's health authority in the same period of cases.[9]

[d] The number of days since the cumulated number of cases was equal to or greater than 100.[10]

[e] Share of the population aged 70 or older

Case growth decomposition

Figure S2 shows how the USA has been moving in a plot similar to Figure 2A. The USA is above the line in the two weeks in which the figure is plotted (exception vis-a-vis other countries).

Figure S3 shows the results of growth decomposition, but with updated data from the week ending April 17th and comparing the (logarithmic) growth vis-à-vis the previous week. Countries and territories tend to be in different positions compared to Figure 2 of the main text, which used data from one week earlier. For example, in Figure 2 (until April 11th), almost all US states were above the zero case growth line. In contrast, in Figure S3B we observe that many states have moved below with heterogeneous combinations of testing and PTR changes.

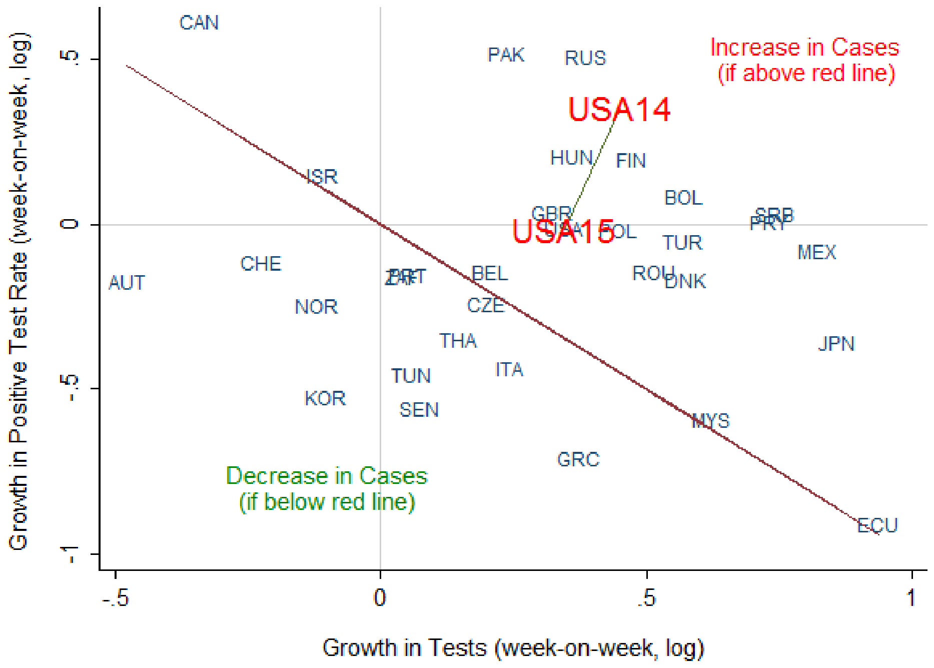

Figure S2. Illustration of weekly growth decomposition in COVID-19 for the USA For the global list of countries, we plot the difference between April 3 through 9 and the previous week, all growth in logarithmic points and per capita. The USA is plotted twice, showing two weekly (log) differences.

a

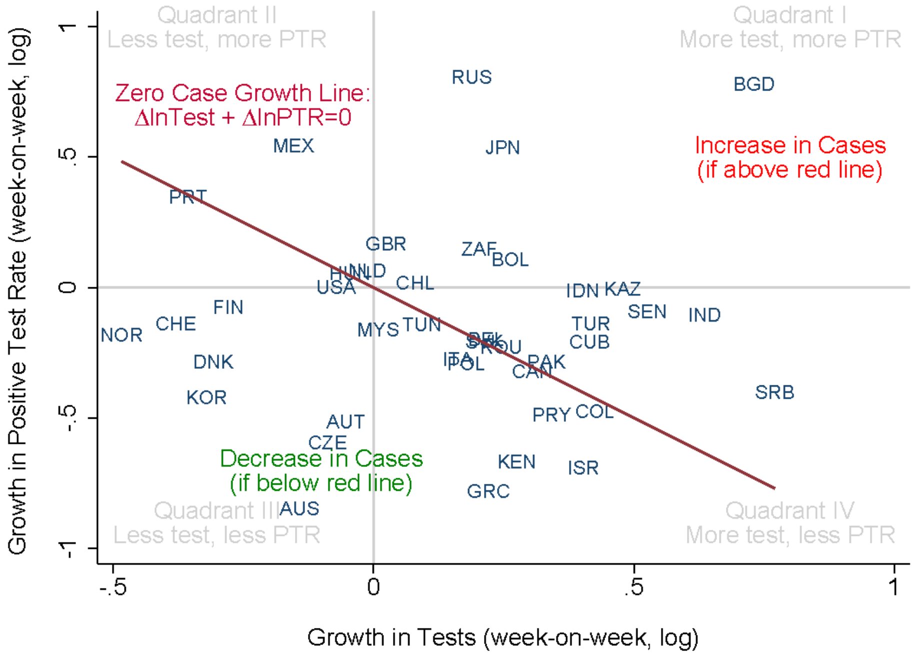

b

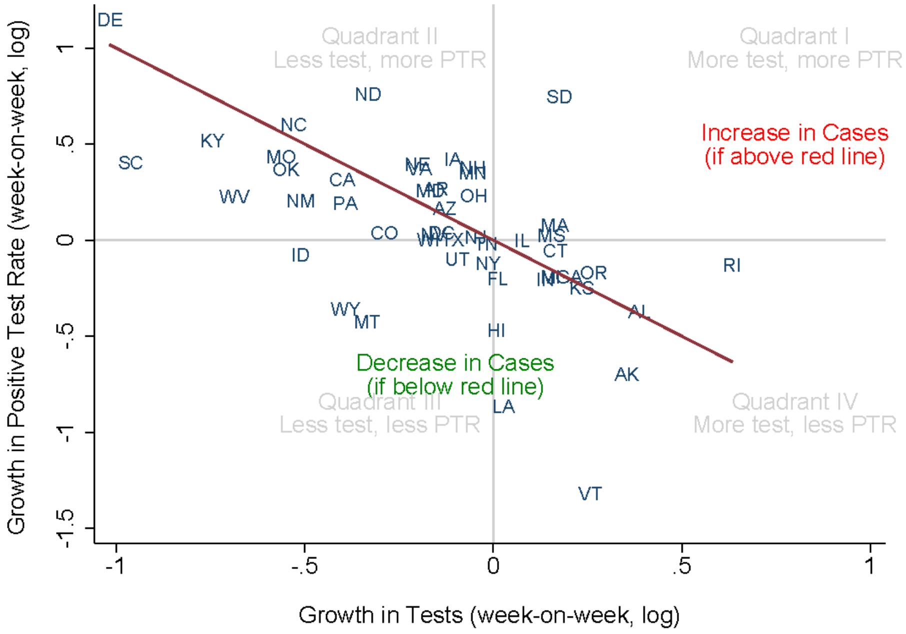

Figure S3. Mapping the Growth Decomposition of Cases: Testing Growth vs. PTR Growth. Week-on-week growth of Testing and PTR across (A) countries and (B) the US States. (Equivalent to Fig 2 of the main text, but updated for the week ending April 17, 2020). Points are countries (A) or US states (B). In both panels, we plot the growth of tests and PTR between the week ending April 17th and the previous week. The functional form of the previously mentioned growth is the change in the natural logarithm of per capita rates per week. The growth decomposition of cases is the sum of the increase in testing (horizontal axis) and PTR growth (vertical axis). The line is not a regression but represents zero case growth as $\Delta \ln Cases \equiv \Delta \ln Tests + \Delta \ln PTR = 0$. While

territories above the line have growing cases, territories below the line have decreasing cases. The four quadrants (I to IV) in gray show the various combinations of increasing or decreasing testing and PTR. Notably, quadrants (II) and (IV) include growing and declining cases, depending on the side of the zero-case growth line. For instance, in (B), KY appears with decreasing cases but is associated with fewer tests and more PTR. The opposite combination happened in (A) for Ecuador. For visual purposes, countries in A were restricted to over 5 million people, and US states in (B) exclude WA. Countries are represented by ISO 3 codes, and the US states by two-digit codes. The Online Technical Appendix shows how the USA moved over several weeks in the cross-country plot.

Elasticities can vary by the country's human development index

Here we show the results from a model with different elasticities for a setting-specific covariate like the country's level of development. In particular, we use the UNDP's Human Development Index as an interaction term. The regression is as follows

$$\Delta \ln Cases_{it} = \beta_{MainEffect} \ \Delta \ln Tests_{it} + \beta_{Interaction} \ \Delta \ln Tests_{it} \times HumanDevel_i + \mu_i + \lambda_t$$

; where $HumanDevel_i$ is country i's Human Development Index demeaned from the global average of that same variable. The demeaning's goal is to reflect the average elasticity on $\beta_{MainEffect}$; while the difference in elasticity due to differences in human development is captured by $\beta_{Interaction}$. Importantly, the non-interacted $HumanDevel_i$ is only included implicitly in the regression, since the country fixed-effect takes care of any time-invariant characteristic, like this index that does not vary within aour period of analysis.

The regression for global data of weekly changes in the second part of the year 2020 displays a statistically significant coefficient $\beta_{Interaction}$ of 1.1 (p-value < 0.001). The figure below (Fig S4) illustrates the practical magnitude of these coefficients. The figure compares the overall elasticity between a country in the 20th percentile of Human Development Index and a country in the 80th percentile. While the former has an overall elasticity of 0.44, the latter's elasticity is 0.71, with 95% confidence intervals that do not overlap. That means that quantitatively, during this period richer countries tended to have stronger elasticities. But qualitatively, both groups of countries have elasticities below one, which are less than proportional. This is our central claim for the week-to-week regressions in the main text.

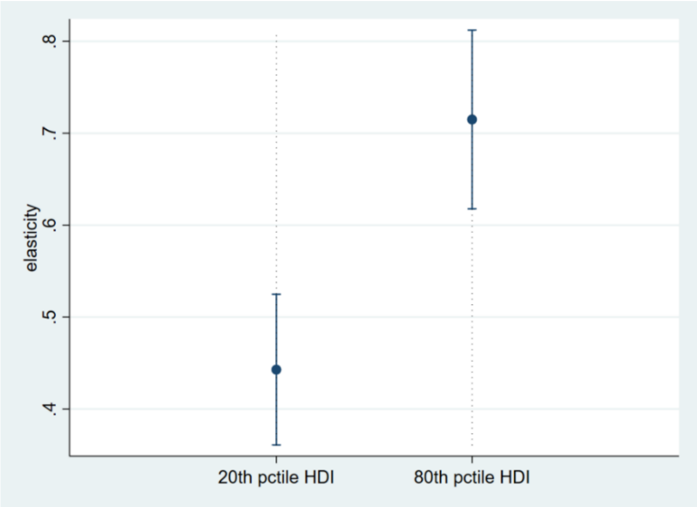

Figure S4. Elasticities estimated for countries at the 20th and 80th percentile of the Human Development Index (HDI). Test elasticity of Covid-19 cases using a weekly frequency from July 1st through December 31st 2020. The figure was estimated from the equation $\Delta \ln Cases_{it} = \beta_{MainEffect} \ \Delta \ln Tests_{it} + \beta_{Interaction} \ \Delta \ln Tests_{it} \times HumanDevel_i + \mu_i + \lambda_t$; and evaluated at HDI corresponding to the 20th and 80th percentiles in the sample.

C. Names and abbreviations for countries and US States

Table S5. Names and abbreviations for countries included in the study

Country and Territory	ISO 3 digit	ISO 2 digit	Country and Territory	ISO 3 digit	ISO 2 digit
Austria	AUT	AT	Lithuania	LTU	LT
Australia	AUS	AU	Latvia	LVA	LV
Bangladesh	BGD	BD	Mexico	MEX	MX
Belgium	BEL	BE	Malaysia	MYS	MY
Bolivia	BOL	BO	Netherlands	NLD	NL
Canada	CAN	CA	Norway	NOR	NO
Switzerland	CHE	CH	New Zealand	NZL	NZ
Colombia	COL	CO	Panama	PAN	PA
Costa_Rica	CRI	CR	Peru	PER	PE
Cuba	CUB	CU	Pakistan	PAK	PK
Czechia	CZE	CZ	Poland	POL	PL
Denmark	DNK	DK	Portugal	PRT	PT
Ecuador	ECU	EC	Paraguay	PRY	PY
Estonia	EST	EE	Romania	ROU	RO
Greece	GRC	EL	Serbia	SRB	RS
Finland	FIN	FI	Russia	RUS	RU
Croatia	HRV	HR	Slovenia	SVN	SI
Hungary	HUN	HU	Senegal	SEN	SN
Indonesia	IDN	ID	Thailand	THA	TH
Israel	ISR	IL	Tunisia	TUN	TN
India	IND	IN	Turkey	TUR	TR
Italy	ITA	IT	United Kingdom	GBR	UK
Japan	JPN	JP	United States	USA	US
South_Korea	KOR	KR	Vietnam	VNM	VN
Kazakhstan	KAZ	KZ	South Africa	ZAF	ZA

Table S5. Names and abbreviations for countries included in the study

State	ISO 2 digit	State	ISO 2 digit
Alaska	AK	Montana	MT
Alabama	AL	North Carolina	NC
Arkansas	AR	North Dakota	ND
Arizona	AZ	Nebraska	NE
California	CA	New Hampshire	NH
Colorado	CO	New Jersey	NJ
Connecticut	CT	New Mexico	NM
District of Columbia	DC	Nevada	NV
Delaware	DE	New York	NY
Florida	FL	Ohio	OH
Georgia	GA	Oklahoma	OK
Hawaii	HI	Oregon	OR
Iowa	IA	Pennsylvania	PA
Idaho	ID	Rhode Island	RI
Illinois	IL	South Carolina	SC
Indiana	IN	South Dakota	SD
Kansas	KS	Tennessee	TN
Kentucky	KY	Texas	TX
Louisiana	LA	Utah	UT
Massachusetts	MA	Virginia	VA
Maryland	MD	Vermont	VT
Maine	ME	Washington	WA
Michigan	MI	Wisconsin	WI
Minnesota	MN	West Virginia	WV
Missouri	MO	Wyoming	WY
Mississippi	MS		

References in the supplementary material

1 European Centre for Disease Prevention and Control. *Data on the geographic distribution of COVID-19 cases worldwide*, <https://bit.ly/2XWhCm2> (2020).

2 The COVID Tracking Project. *State by state data and annotations*, <https://covidtracking.com/data> (2020).

3 Roser, M., Ritchie, H., Ortiz-Ospina, E. & Hasell, J. *Coronavirus Pandemic (COVID-19)*, <https://bit.ly/2ROHdts> (2020).

4 Centers for Medicare and Medicaid Services. *Health expenditures by state of residence, 1991-2014*, <https://go.cms.gov/2KkMk0f> (2017).

5 World Health Organization. *Global Health Expenditure Database*, <https://apps.who.int/nha/database> (2020).

6 James, S. L. *et al.* Global, regional, and national incidence, prevalence, and years lived with disability for 354 diseases and injuries for 195 countries and territories, 1990-2017: a systematic analysis for the Global Burden of Disease Study 2017. *The Lancet* **392**, 1789-1858, doi:10.1016/S0140-6736(18)32279-7 (2018).

7 Murray, C. J. L. *et al.* Population and fertility by age and sex for 195 countries and territories, 1950-2017: a systematic analysis for the Global Burden of Disease Study 2017. *The Lancet* **392**, 1995-2051, doi:10.1016/S0140-6736(18)32278-5 (2018).

8 Hawawini, G. Why beta shifts as the return interval changes. *Financial analysts journal* **39**, 73-77 (1983).

9 Hasell, J. *et al.* A cross-country database of COVID-19 testing. *Scientific data* **7**, 345 (2020).

10 Dong, E., Du, H. & Gardner, L. An interactive web-based dashboard to track COVID-19 in real time. *The Lancet Infectious Diseases* **20**, P533-P534 (2020).
